# The demographics of human and malaria movement and migration patterns in East Africa

**DOI:** 10.1186/1475-2875-12-397

**Published:** 2013-11-05

**Authors:** Deepa K Pindolia, Andres J Garcia, Zhuojie Huang, David L Smith, Victor A Alegana, Abdisalan M Noor, Robert W Snow, Andrew J Tatem

**Affiliations:** 1 Emerging Pathogens Institute, University of Florida, Gainesville, FL, USA; 2 Department of Geography, University of Florida, Gainesville, FL, USA; 3 Malaria Public Health Department, KEMRI-Wellcome Trust-University of Oxford Collaborative Programme, Nairobi, Kenya; 4 Center for Infectious Disease Dynamics, Pennsylvania State University, Pennsylvania, USA; 5 Department of Biology, Pennsylvania State University, Pennsylvania, USA; 6 Department of Epidemiology, Johns Hopkins Bloomberg School of Public Health, Baltimore, USA; 7 Fogarty International Centre, National Institutes of Health, Bethesda, MD 20892, USA; 8 Center for Disease Dynamics, Economics and Policy, Washington DC, USA; 9 Department of Geography and Environment, University of Southampton, Southampton, UK; 10 Centre for Tropical Medicine, Nuffield Department of Clinical Medicine, University of Oxford, Oxford, UK

## Abstract

**Introduction:**

The quantification of parasite movements can provide valuable information for control strategy planning across all transmission intensities. Mobile parasite carrying individuals can instigate transmission in receptive areas, spread drug resistant strains and reduce the effectiveness of control strategies. The identification of mobile demographic groups, their routes of travel and how these movements connect differing transmission zones, potentially enables limited resources for interventions to be efficiently targeted over space, time and populations.

**Methods:**

National population censuses and household surveys provide individual-level migration, travel, and other data relevant for understanding malaria movement patterns. Together with existing spatially referenced malaria data and mathematical models, network analysis techniques were used to quantify the demographics of human and malaria movement patterns in Kenya, Uganda and Tanzania. Movement networks were developed based on connectivity and magnitudes of flow within each country and compared to assess relative differences between regions and demographic groups. Additional malaria-relevant characteristics, such as short-term travel and bed net use, were also examined.

**Results:**

Patterns of human and malaria movements varied between demographic groups, within country regions and between countries. Migration rates were highest in 20–30 year olds in all three countries, but when accounting for malaria prevalence, movements in the 10–20 year age group became more important. Different age and sex groups also exhibited substantial variations in terms of the most likely sources, sinks and routes of migration and malaria movement, as well as risk factors for infection, such as short-term travel and bed net use.

**Conclusion:**

Census and survey data, together with spatially referenced malaria data, GIS and network analysis tools, can be valuable for identifying, mapping and quantifying regional connectivities and the mobility of different demographic groups. Demographically-stratified HPM and malaria movement estimates can provide quantitative evidence to inform the design of more efficient intervention and surveillance strategies that are targeted to specific regions and population groups.

## Background

Increased investment in malaria control and international donor support in recent years has led to reductions in transmission, morbidity and mortality in many malaria endemic parts of the world [1-4]. The movement of malaria parasites, primarily through the movement of infected humans, is important for successful intervention strategies across the full range of transmission intensities. Human population movement (HPM) from higher transmission areas risks reintroduction and resurgence in malaria-free receptive areas, and has undermined elimination efforts in the past [5-8]. In non-elimination settings, understanding the patterns of parasite dispersal from local hotspots of transmission can aid the design of additional targeted control by identifying both the regions where imported infections originate and where they may contribute substantially to transmission [9]. Finally, HPM patterns have driven the spread of drug resistant parasite strains [10,11]. Strategic control and elimination plans should therefore be built on a strong evidence base including information on HPM and likely parasite movement volumes and routes [9]. Moreover, identifying key demographic groups most likely to carry infections can provide useful information for tailored and targeted intervention and surveillance efforts [12].

A variety of data types, statistical analyses and mathematical models have been used to quantify HPM patterns [13,14] and specific HPM patterns relevant for malaria dynamics [15-20] at different spatial scales. National surveillance data, such as hospital patient records, that provide individual-level travel history and demographic data have also been used to directly quantify features of imported malaria cases [21]. However, surveillance data is likely to miss asymptomatic parasite carriers and non-health seeking cases [22], and comprehensive and reliable surveillance systems to detect imported cases are generally under developed in low-income countries. In these settings, directly estimating malaria movement has primarily been based on travel history data from selected population groups or geographic areas, with travel studied as a possible risk factor for infection [23,24]. Recently however, the availability of various HPM data types, high resolution spatially-referenced *Plasmodium falciparum* and *Plasmodium vivax* malaria metric data [25,26], mathematical models [27-30] and computational tools have provided an alternative approach to indirectly measure malaria movements [18]. Airline passenger networks and *P. falciparum* malaria transmission maps been used to model large-scale malaria movements [31,32]. Novel study methods based on mobile phone usage data combined with *P. falciparum* malaria transmission maps, for example, have begun to tackle HPM and malaria movement dynamics at other scales [19], such as in Zanzibar island and at a national level in Kenya [9,33]. Demographic and socioeconomic breakdowns of HPM, personal malaria protection and motivations for HPM have been reviewed in the context of malaria control and elimination [18]. However, detailed comparisons of high-risk demographic HPM groups have not been undertaken at a national or regional level, despite their importance in understanding malaria movement and refining quantitative evidence for guiding policy decisions.

National population and housing census data can provide valuable individual-level records for quantifying migration, travel and connectivity at a national scale, that have been shown to correlate strongly to finer temporal scale HPM [34], and have been used to analyse migration patterns for many years [35-37]. Census and national household surveys also provide individual-level data for demographic and socioeconomic characteristics, motivations for travel and use of bed nets, which if analysed in a systematic way, can be used to illustrate relative HPM variations within the population [38]. Here we collate HPM datasets from Kenya, Uganda and Tanzania and use them with network analysis techniques and Geographical Information Systems (GIS) tools to describe and examine HPM patterns across differing spatiotemporal scales and demographic groups. By combining these data with *P. falciparum* transmission maps and mathematical models, the demographic groups most likely to move and carry infection were explored, and likely sources, sinks, connectivity and importation routes of infection-carrying individuals compared between demographic groups. Finally, within group heterogeneities in short-term travel and bed net use were assessed to further illustrate the heterogeneities in travel and risk patterns that exist, and to identify high-risk malaria movement groups.

## Methods

### Data

#### *Human population movement (HPM) data*

Migration and movement data from national household survey data and national statistical bureaus for Kenya, Uganda and Tanzania were obtained (Additional file 1). Individual-level census and survey data that included HPM data (migration and short-term travel-related questions), demographic descriptions, rural-urban stratifications and malaria-relevant records (such as bed net use) were obtained. The available census and survey datasets differed in terms of sample sizes, represented populations, migration and travel questions asked, rural and urban location records, demographics captured and malaria-relevant variables recorded. However, datasets were similar in the way that HPM, demographic characteristics and rural-urban status were defined. HPM was defined in two ways. First, an individual origin-destination specific migration (described as a flow between first and/or second-level administrative boundaries within a country) was identified if the previous residence location of an individual differed from current residence location. Second, short-term travel per individual was identified if the individual spent time away from their current place of residence. Demographic characteristics were described by age and gender. Rural-urban status was defined for each individual based on the rural-urban status of residence households. Individual bed net use was assessed based on whether an individual slept under a bed net the night before the data collection date.

#### *National census migration micro-data*

Census micro-data, a systematically selected subset of countrywide national housing and population census data obtained from the Integrated Public Use Microdata Series (IPUMS) [39], was obtained for all three countries. The census micro-data for Kenya was a 5% sample from its 1999 census, for Uganda a 10% sample from its 2002 census and for Tanzania a 10% sample from its 2002 census. The data samples included individual-level data for all questions included in each census. Questions about migration, migrants, and their demographics were extracted from the most recent IPUMS data available for each country. IPUMS migration data for Kenya were obtained from current residence districts compared to residence districts 1 year prior to the census (69 administrative level 2 units). For Uganda, migration data were obtained from current residence districts compared to previous residence districts (56 administrative level 2 units) and number of years in current residence districts. For Tanzania, migration data were obtained at a lower spatial resolution than Kenya and Uganda. Previous region (25 administrative level 1 units) of residence was compared to current region of residence to describe migration. Individual-level demographic records (age and gender) and rural/urban status of current residence locations were available for all migrants in all three census micro-data samples.

#### *Migration and short-term travel data from national household surveys*

National household survey datasets with individual-level HPM, demographic and bed net use data were obtained for Kenya. The Kenya Integrated Household Budget Survey (KIHBS) 2004/2005 data was obtained from the Kenya National Bureau of Statistics. Individual-level migration data included current district of residence (same 69 districts as the Kenya 1999 census), district of birth and rural/urban status of current and previous districts. For each individual in the survey, information on age and gender were included. Migration was defined using current district of residence and district of birth, and further stratified by rural/urban location and demographic characteristics. Individual records on the number of cumulative months each individual spent away from home in the past 12 months were used as a proxy for an individual engaging in short-term travel (>1 month away). Other malaria relevant data extracted included bed net use in migrant and non-migrant groups.

#### *Malaria data*

Country-level malaria transmission maps for Kenya, Uganda and Tanzania were obtained from the 2010 global *P. falciparum* endemicity maps (with *P. falciparum* parasite rate standardized for 2-10 year olds (*Pf*PR_2-10_), for 1×1 km pixels) from the Malaria Atlas Project (MAP) [25]. Previously developed mathematical models were used to estimate age-specific *Pf*PR for each administrative unit based on the mean *Pf*PR_2-10_ estimates per administrative unit [29]. Administrative units were grouped into 3 control-relevant endemicity classes: 0 > PfPR_2-10_ ≤ 5, 5 > PfPR_2-10_ ≤ 40 and PfPR_2-10_ > 40 [40].

### Analysis

Census and survey HPM data were extracted and stored in a standardized format representing origin-destination migrant flows (the origins and destinations were defined according to the respective administrative units available in each dataset) and origin-specific short-term flows (origin was defined by current administrative unit of residence). The datasets were organized into categories that represented: type of dataset used to quantify it (census data or survey data), country that the dataset represented, data collection time (for example, month/year in which data was collected), spatial-temporal category of HPM records (migration or short-term travel). Migration data (stratified by age, gender, rural-urban status of destination) was obtained from IPUMS samples for each country and used to undertake between country comparisons. As the definition of a migrant differed between countries, patterns of relative differences within countries were compared, rather than absolute value comparisons (Additional file 2). For Kenya, the IPUMS data was supplemented with household survey data (KIHBS) to compare short-term travel and bed net use immigrant and non-migrant populations, stratified by age and gender. Comparisons were further verified using simple linear regressions (Additional file 3).

#### *HPM and malaria movement networks*

I-PUMS migration data were used to construct stratified migrant flow networks, with origin and destination administrative units for migrant flows as nodes in each network, as previously developed by Tatem *et al. *[41]. Migrant flows were stratified based on age and gender characteristics of individuals and rural/urban status of their destination. The stratified origin-destination migrant flow data were then used to develop three types of stratified directed migrant networks, based on attributes assigned to each network edge: (i) un-weighted migrant networks, where directional migrant flow between each origin-destination pair existed if at least one individual HPM was recorded, (ii) weighted migrant networks, where the magnitude of directional migrant flows between each origin-destination pair formed the edge weights of each network and (iii) malaria movement networks, where the edge weight represented directional migrant flows weighted by the mean age-specific *Pf*PR at the origin. Migrants from higher endemicity areas had a larger weighting than migrants from lower endemicity areas, based on their current age. Larger weights represented high likelihoods of malaria movement between location pairs.

Network analysis tools in the R igraph package [42] were used to identify and compare key features and properties of each stratified network and make relative comparisons between countries and demographic groups. Local and global network measures were used to quantify and compare structural characteristics of networks and of nodes within networks [43]. Local network measures were used to examine node-specific (location-specific) centrality within each country-level network and make comparisons between locations within a specific network included measures for un-weighted networks (in, out and total node degree, which represented the number of inward/outward/total connections to node) and weighted networks (in/out/total strength, which represented the number of inward/outward/total flows to a node). Local network measures were summarized for each stratified network to examine and compare global structure and characteristics of networks.

Migration patterns (connectivity), represented by un-weighted directional networks, were compared between demographic and rural-urban groups using mean degree (mean number of per location connections) for each network. Weighted migration networks were compared to assess relative differences in migration magnitudes, using mean strength of the HPM flow networks (mean number of migrant flows per location). Malaria movement networks were compared to assess relative differences in likely malaria movement magnitudes, using mean strength of the malaria movement networks (mean number of malaria-weighted migrant flows per location). Weighted migrant networks were compared to weighted malaria movement networks to illustrate changes in the relative strength of ties within stratified networks, to give an indication of the demographic groups most likely to carry infections. As migration data differed between countries (Additional file 2), mean network measures were standardized by the respective total for each measure to make comparisons between countries. Non-parametric multiple comparison Kruskal-Wallis tests were used to compare standardized mean network connectivity, mean HPM strength and mean malaria movement between age, gender, rural-urban groups for each country and boxplots were used to address within age group variations (Additional file 4).

#### *HPM and malaria-weighted sources and sinks*

Most likely 'sources’ (districts/regions more likely to export migrants and malaria movements), 'sinks’ (districts/regions more likely to import migrants and malaria movements) and routes of migrant flows and malaria movements, stratified by age and gender of migrants and rural-urban status of destinations were identified. Migrant sources and sinks were identified by the number of outward and inward migrants per administrative location, respectively. Outward malaria movement was estimated by weighting migrant flows by origin endemicity. Weighted estimates were obtained by multiplying age-specific outflows by age-specific mean *Pf*PR for each origin (Index 1). Locations with low mean endemicity but large migrant outflows were comparable to high mean endemicity origins with fewer migrant outflows if index values were similar. Low index values represented administrative units with low endemicity and few HPM outflows. Incoming malaria movement was obtained by multiplying each origin-specific migrant inflow by age-specific *Pf*PR at the origin, for each destination (Index 2). Locations with large migrant inflows from low endemicity origins were comparable to locations with fewer migrant inflows from high endemicity origins. The most likely sources and sinks of HPM and malaria movements were mapped and compared between age groups to illustrate relative differences between demographic groups in each country. 10–20 and 20–30 year age groups were contrasted in detail to illustrate similarities and differences.

**Index 1:** Outgoing malaria-weighted flows from district j, to all other districts i

$$\mathrm{agePf}\mathrm{PRj}*\Sigma_{j=1}^{n}\text{age}\mathrm{HPMij}$$

**Index 2:** Incoming malaria-weighted flows to district i, from all other districts j

$$\Sigma_{i=1}^{n}\mathrm{agePf}\mathrm{PRi}*\text{age}\mathrm{HPMij}$$

#### *HPM and malaria-weighted flows*

Origin-destination specific HPM flow networks, stratified by age, gender and rural/urban status were represented as directional network flow maps. The network maps were developed to show whether incoming migrants and malaria movements originated from specific parts of the country or whether origins were dispersed between various places. The age-stratified flow maps were overlaid on categorized mean age-specific *Pf*PR maps to illustrate flows between different transmission zones [40] in each country. To illustrate the most important age-specific malaria movement routes in each country, origin-destination location pairs were ranked by weighting HPM flow by the mean age-specific *Pf*PR at the origin, for each origin-destination pair (Index 3). Origin-destination pairs with the largest origin endemicity and the largest flows between them were assumed to be the most likely routes for infection flow. The 10 most important HPM and malaria movement flow routes for each country were compared to illustrate relative differences in malaria movement between age groups. The 10–20 and 20–30 year age groups were again contrasted in detail to illustrate similarities and differences.

**Index 3:** Ranking directional origin-destination specific HPM as relevant for likely malaria movement

agePfPRi*ageHPMij

#### *Short-term travel and bed net use immigrant and non-migrant populations*

Short-term travel and bed net use, stratified by age and gender, were compared between migrant and non-migrant populations in Kenya, by first plotting the data, then verifying the differences using linear regression (Additional file 3). Short-term travel by immigrants was compared between age and gender groups, stratified by rural-urban status to assess travel and bed net use in high-risk migrant groups. Short-term travel was then compared between migrant and non-migrant groups. Individual bed net use was compared within migrant groups and between immigrants and non-migrants, as stratified by age group and gender.

All raw data sets were extracted, reformatted and stored using Microsoft Excel, Microsoft Access and R.

HPM data was extracted and organized using Microsoft Excel, Microsoft Access SQL queries and R. Malaria endemicity data was analysed in ArcGIS and R. Network methods were applied using the igraph package in R. Multiple comparison Kruskal-Wallis tests were implemented in R [44].

## Results

### HPM and malaria movement networks

**Figure 1:**
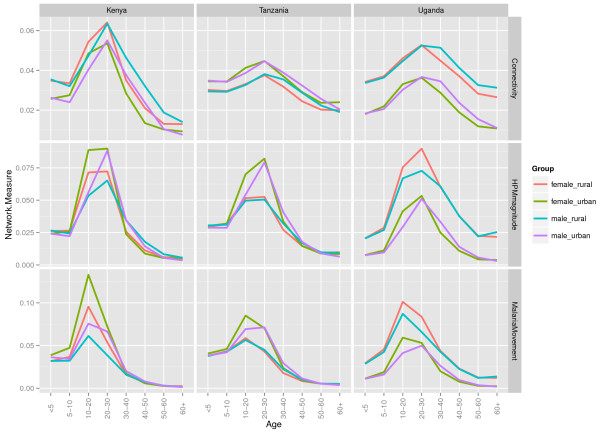
**Comparing patterns and magnitudes of human population movement (HPM) and malaria movement in East Africa, based on mean degree and network strength measures, for each age, gender and rural-urban stratified network.** Mean degree was used to measure differences in HPM connectivity and strength was used to measure magnitudes of HPM flow and likely malaria movement.

Migrant and malaria networks in East Africa showed various differences and similarities in patterns and magnitudes between countries, age groups, genders and urban/rural settings (Figure 1, Additional files 2 and 4).

When examining the differences in connectivity of migrant networks between age groups (measured using mean network degree), the 20–30 year old age group had the highest values (Figure 1, 1st row of graphs), illustrating that this age group was likely to migrate between the largest variety locations within each country. Further heterogeneity in connectivity was seen when networks were stratified by gender. For older age groups, connectivity was higher amongst males, and for younger age groups, female migrant networks were more connected. Age and gender connectivity differences were not uniform between countries. For example, within the younger age groups, <5 years olds were shown to have higher connectivity compared to 5–10 year olds in Kenya, indicating that young children may be likely to migrate with their parents, which was not evident in the Uganda and Tanzania data. Rural-urban differences in connectivity were also revealed, showing that migrants currently living in rural areas had significantly higher connectivity than migrants living in urban areas in Uganda (based on the Kruskal-Wallis test - Additional file 4). However, in Kenya and Tanzania, urban resident migrant connectivity was higher.

Magnitudes of migrant flows (measured using mean network strength) were highest in the 20–30 year old age group (Figure 1, 2nd row of graphs), showing similar results to connectivity. With more female internal migrants than males in all three countries, the overall patterns in the region were dominated by females. Gender-specific and rural-urban migration flow heterogeneities were also seen. In Uganda, overall magnitudes of migrant flows were higher for rural migrants for all age groups, however gender differences were only seen in the 20–30 year age group. For Kenya and Tanzania, differences between rural and urban migrants were only seen for the 10–20 and 20–30 year old age groups and not for younger or older migrants.

When migrant networks were weighted by origin age-specific mean *Pf*PR, peak flow magnitudes shifted to the 10–20 year old age group for all age, gender and rural-urban stratifications, except Ugandan and Tanzanian urban males (Figure 1, 3rd row of graphs). As with connectivity and migration magnitudes, malaria movements differed between gender and rural-urban stratifications. In Kenya, females were more likely to import malaria compared to males for ages less than 20 years. This meant that in general, origins of female migrants younger than 20 years old had higher mean age-specific *Pf*PR than origins of male migrants in the same age group. In Tanzania, urban migrants aged 10–40 years had larger malaria-weighted flows than rural-residing migrants. However, in Uganda, magnitudes of malaria-weighted flows were always higher in rural-residing migrants than urban-residing migrants.

Statistically significant differences between mean connectivity and mean strength of each stratified network, based on the Kruskal-Wallis test, were detected for age, gender and rural-urban stratified networks for all three countries. Within age group variation in connectivity, migration magnitudes and relative malaria movements were also detected and differed between countries (Additional file 4).

### Source and sinks of HPM and malaria movement

**Figure 2:**
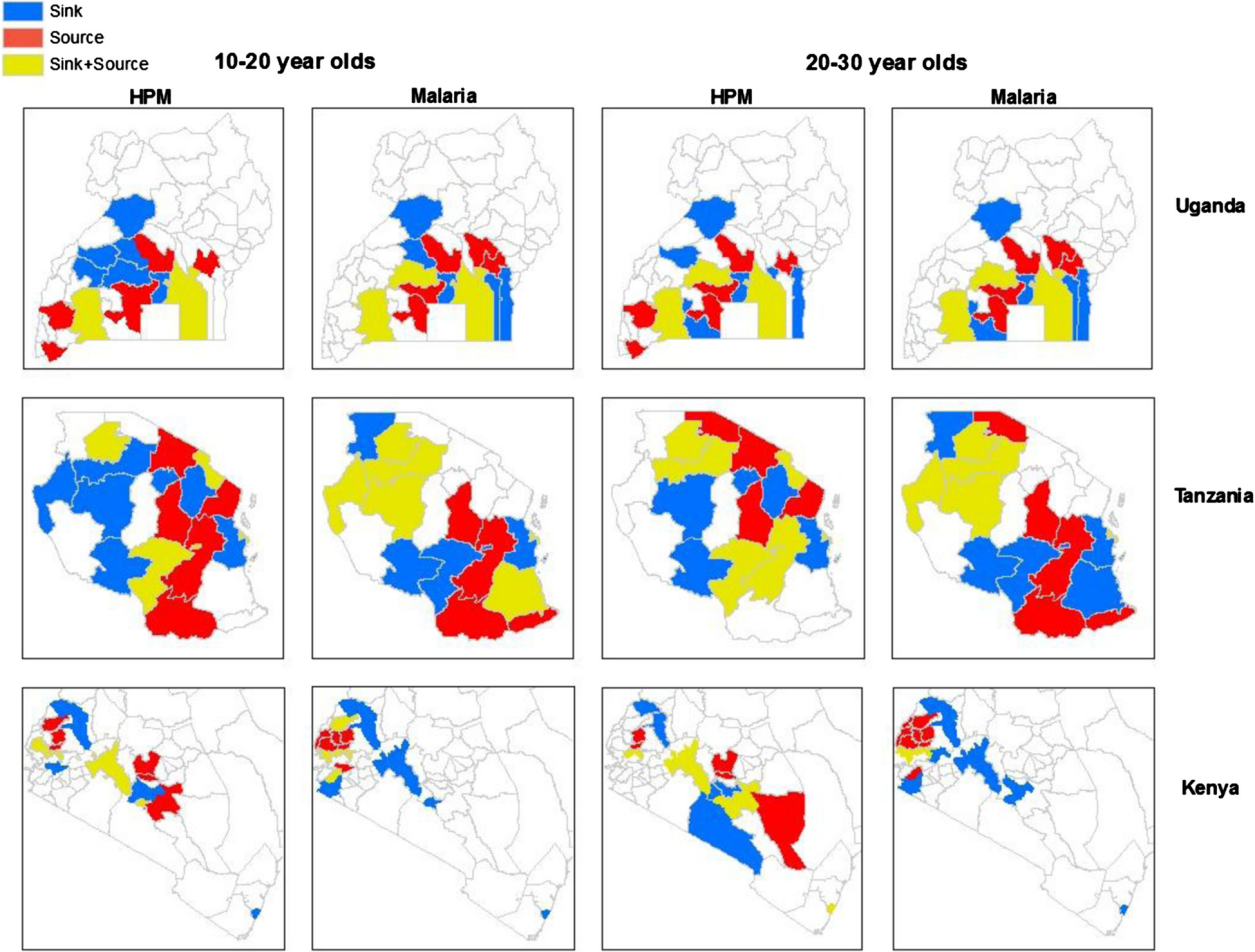
**Comparing age-stratified human population movement (HPM) and likely malaria movement sources and sinks in East Africa.** The ten most likely age-specific sources of HPM and likely malaria movements, which represent districts/regions that were most likely to export HPM and malaria, are coloured in red. The ten most likely age-specific sinks of HPM and likely malaria movement, which represent districts/regions that were most likely to import HPM and malaria, are coloured in blue. The ten most likely districts/regions that were likely to both export and import HPM and malaria are coloured in yellow.

Within each country, some districts/regions were more likely to be sources, whilst others were more likely to be sinks for migrant flows and malaria movements (Figure 2). The overall spatial distribution of sources and sinks differed between HPM and malaria movements and between countries. In Kenya, HPM sources were more spread out in the southern part of the country, however, sources of malaria movement were concentrated around the Lake Victoria and western region. As seen for Kenya, malaria movement sources were also close to the Lake Victoria region in Uganda. Tanzania showed a different pattern from Uganda and Kenya, with sources and sinks of HPM and malaria movement distributed across the country. Some districts ranked high as HPM sources/sinks, but were not as important for malaria movement. For example, in Tanzania, northern regions were likely sources of HPM but unlikely sources of imported infections.

The spatial distribution of most likely sources and sinks of HPM and malaria movement also differed between demographic groups (10–20 and 20–30 year age groups compared in Figure 2). Some districts/regions were important in both importing and exporting HPM and malaria, and this also differed between age groups. For example, Nairobi was both a source and sink of HPM (according to IPUMS 1999 data, 16.5% of Nairobi’s population had moved from another district) and sink for malaria movement in both 10–20 and 20–30 year old age groups. Mombasa, the second largest city in Kenya, was a sink for HPM and malaria movement for both age groups, and was also a source for HPM in the 20–30 year old age group. Unlike in Kenya, Dar es Salaam, the commercial capital of Tanzania, was a sink and a source for both HPM and imported infections, and the region in which Dodoma, the national capital, is located was only a source of HPM and malaria movement and not a sink. Similarly, Kampala, Uganda’s capital city, was a source and sink for both HPM and malaria movement. Overall, HPM source/sink patterns were different between the 10–20 and 20–30 year old age groups, however, malaria movement patterns were more similar between age groups for Uganda and Tanzania, but not Kenya, at a national scale.

### HPM and malaria-weighted flows

**Figure 3:**
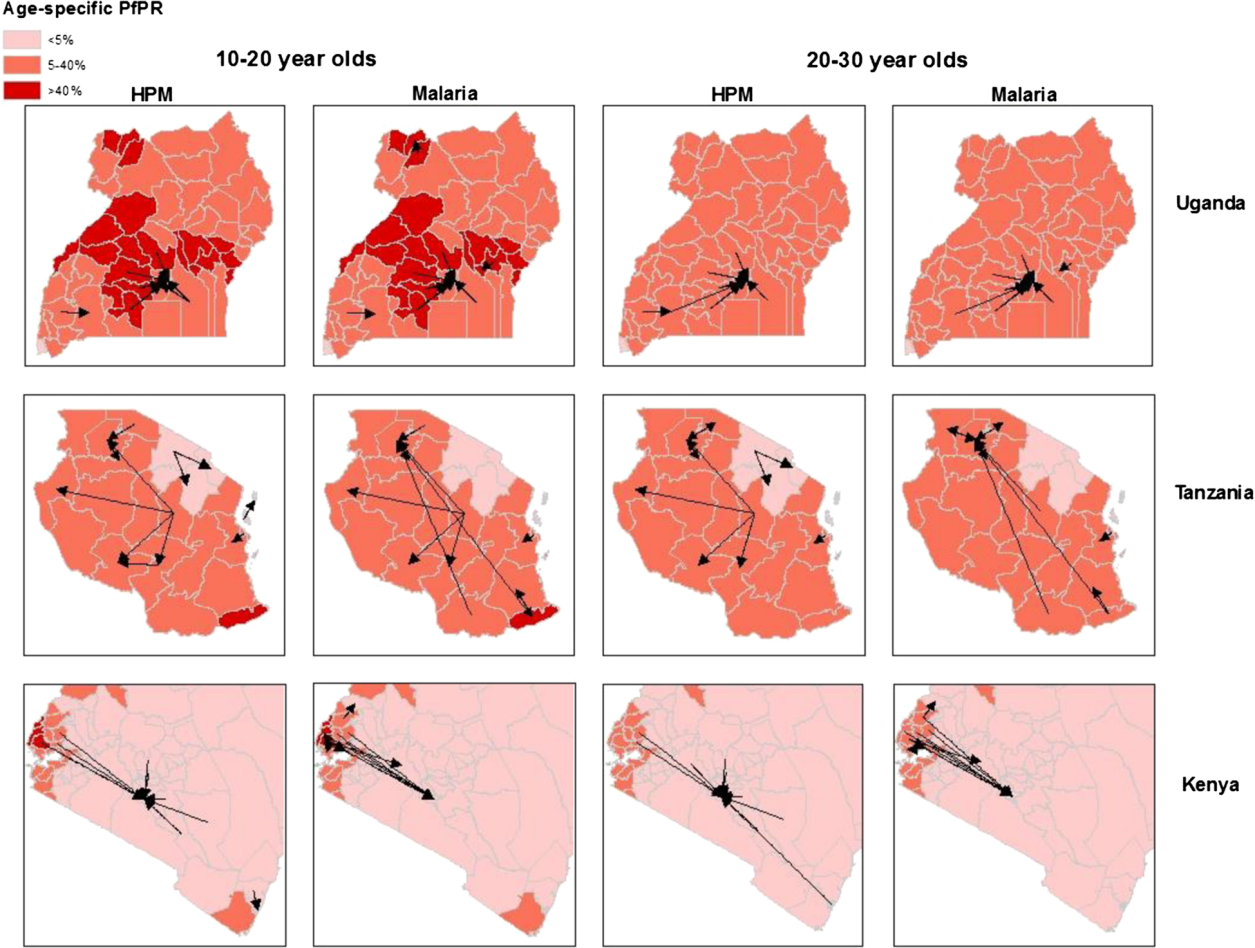
**Comparing age-stratified human population movement (HPM) and likely malaria movement flow routes in East Africa.** Arrows represent directional HPM flows and likely malaria movements between districts/regions. Flows are overlaid on categorized age-specific mean *Pf*PR maps to illustrate HPM between transmission zones.

The most common routes for HPM flows and the most likely routes for malaria movement were different between demographic groups for all three countries (Figure 3). In Kenya, Nairobi was a major sink of HPM and malaria movements whilst the Lake Victoria region was a major source (Figure 2). The flow maps showed that origins of HPM into Nairobi were likely to be various parts of the country, and this differed between age groups, however, malaria movements primarily originated in the Lake Victoria region for both age groups (Figure 3). In Uganda, Kampala was both a source and sink for HPM and malaria, however top ranking origin-destination specific HPM and malaria movement flows were into Kampala from surrounding districts for both age groups. In Tanzania, both HPM and likely malaria movement routes occurred over large distances (relative to Kenya and Uganda). The region in which Dodoma is located was a major source of HPM and malaria movement (Figure 2), with the largest migrant outflows to central and western parts of the country for both age groups. For malaria movements in the 20–30 year old age group however, Dodoma was a top ranking source of malaria movements specifically to the northern region in the Lake Victoria area, where Mwanza city is located.

### Short-term travel and bed net use immigrant and non-migrant populations

**Figure 4:**
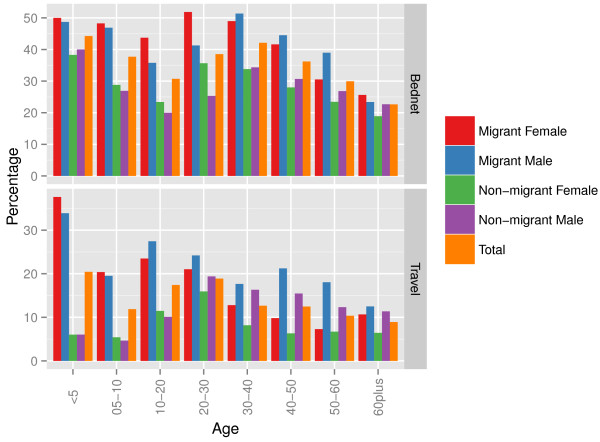
**Comparing bed net use and short-term travel between migrants and non-migrants, stratified by age and gender in Kenya.** For each age group, the proportion of individuals that slept under a bed net the previous night, out of all individuals enumerated, was obtained from the Kenya Integrated Household Budget Survey (KIHBS). Similarly, for each age group, the proportion of travellers from all individuals enumerated was obtained.

Short-term travel and bed net use differed within migrant groups and between immigrants and non-migrants (Figure 4, Additional file 3). Within migrant groups, the highest proportions of travellers were <5 years old, followed by 10–20 year olds. In general, short-term travel in younger immigrant females (<5 and 5–10 year olds) was more likely than in immigrant males, but for older age groups short-term travel was more likely in males. It is important to emphasize that the 10–20 year old age group was estimated to have the highest likelihoods of malaria movement compared to other age groups, along with relatively high likelihoods of short-term travel. Differences were also seen in travel patterns when age-stratified migrants were further stratified by rural-urban status of previous (origin) residence (Additional file 3). Bed net usage was higher in female immigrants compared to males for younger age groups, and higher for males in older age groups. Bed net use in the 10–20 year old age group was relatively low compared to other age groups, further emphasizing the importance of this age group in malaria movement. When comparing migrants to non-migrants, migrants were more likely to engage in short-term travel (recorded as having been away from normal residence at least once) than non-migrants for all age and gender groups. The largest differences in short-term travel between migrants and non-migrants were seen in children (<5, 5–10 and 10–20 year age groups) for both males and females. Immigrant groups, those of <20 years were more likely to travel than older age groups, however in non-migrant groups, 20–30 year olds were most likely to engage in short-term travel. Differences were also seen in bed net use between migrants and non-migrants (Additional file 3).

## Discussion

The quantification and analysis of HPM can be important for successful planning of both malaria control and elimination [45]. As shown here, patterns and magnitudes of HPM and individual infection rates differ between demographic groups [29], and further differences in individual behaviors, such as bed net use and short-term travel [18], lead to variation in likelihoods of malaria movement between these sub-population groups. Quantifying these differences allows identification of key demographic groups, and the likely sources, sinks and routes of infection inflow [9], which enables the development of more stringent surveillance systems, through prioritizing data collection and targeting resources to populations and areas where imported infections are likely [12,46]. Identifying high-risk immigrants may also pin-point where drug resistance strains may arise or spread [11] and enable more efficient targeting of effective anti-malarial treatments. In areas of heterogeneous transmission risk, local hotspots can provide specific targets for strategic intervention deployment [47,48]. Within these hotspots, identifying high-risk demographic groups likely to import and export infections and how they connect other transmission zones across a country may enable further refinement of intervention targets and development of cost-efficient attack strategies. For residents of low transmission areas traveling to hotspots, for example, in the context of boarding school children, providing prophylaxis before children travel to higher endemicity home locations, insecticide spraying in dormitories and provision of bed nets may be adequate measures. On a larger scale, education programmes targeted at high-risk mobile populations or regions identified here may also ensure bed net usage and treatment seeking rates become higher in these key groups.

Due to varying infection rates and the malaria endemicity at the place of origin between age groups [29], those age groups that exhibit higher movement rates may not be those with the highest infection prevalence (Figure 1). Here it was shown that in general, the highest rates of HPM are in the 20–30 year age group, as expected with high rates of adult rural–urban migration in low income nations [49], however, likely malaria movements were highest in 10–20 year olds (Figure 1). With the East African culture of boarding school attendance [50], adolescents are likely to migrate between transmission zones, and engage in short-term travel to visit families thereafter (Figure 4). Short-term movements between different transmission zones are likely to be more relevant for estimating numbers of imported infections [9,19], with imported infections into receptive areas threatening local transmission. Migratory moves have however been shown to correlate strongly with shorter term connectivity [34], therefore, quantifying relative differences in demographically stratified migratory moves, as undertaken here, provides a good indication of the relative magnitudes and directions of shorter term HPM patterns. However, these results also showed that bed net use was higher immigrant populations, which may reduce onward transmission of imported infections through short-term travel. With Uganda’s population and geography being largely rural, and major urban centers being smaller compared to Kenya and Tanzania [51], rural HPM and malaria movements dominated urban ones. Implications of imported infections in rural compared to urban areas differ, for example rural areas may generally have higher receptivity, increasing the likelihood of onward transmission [52]. By stratifying HPM and malaria movement by origin-destination descriptions, areas that are most likely to import and export migrants and subsequent malaria movements can be identified (Figure 2). Moreover, migration maps (Figure 3) can help to identify differences in connectivity between demographic groups. In Kenya, high levels of malaria movement connectivity is seen between Nairobi and the Lake Victoria region, matching previous findings [9]. Further stratification of these movements, such as by rural-urban and demographic descriptions, can help define high-risk groups important for urban malaria control. For example, adult male migrants in Nairobi may work on construction sites where pools of stagnant water provide environments for mosquito breeding [53] and imported infections may instigate outbreaks. Defining such high-risk populations may become important in the future if successful control leads to low transmission, driving epidemiological shifts that make adult males more important than traditionally vulnerable pregnant women and children. These alternative high-risk groups that may become a priority when aiming for elimination have recently been termed 'hotpops’ [12,54].

While the results outlined here point to clear patterns and trends, a range of uncertainties still remain. HPM is the most difficult component to measure in the demographic equation [55], and data on it directly captures only long temporal scales of human movement, which may not be the dominant type of movement for the carriage of infections [15]. Nevertheless, it is strongly indicative of shorter temporal scale movements across sub-national spatial scales [34], and thus does provide a valuable indicator of connectivity amongst different demographic groups and regions. Census data does not allow for origin-destination specific within administrative boundary HPM to be estimated. Therefore, even with high-resolution malaria maps, infection heterogeneities at small scales cannot be assessed. Additionally, differently posed migration questions and differences in missing data samples across census datasets limit the use of census data in providing relative comparisons of HPM and malaria movement between countries. In the survey data used here, short-term travel data did not include destinations of travel or durations of stay, which if available could be used with existing mathematical models to estimate individual probabilities of infection acquisition and numbers of imported infections [19,30,33], providing absolute rather than relative measures. Further temporal descriptions may also highlight seasonal variations in travel patterns, which if associated with malaria data, may illustrate seasonal differences in malaria movement. It is important to note however, that limitations do not only exist in mobility and movement data, but also in the biological knowledge of malaria infection acquisition [33], the use of *Pf*PR as a measure for endemicity across transmission intensities [40] and demographic groups, and the difficulty in measuring innate transmission risk, or receptivity, of an area.

Future work will examine the potential of linking the data sources and analyses presented here with other mobility datasets, such as mobile phone usage data [9], to estimate demographically-specific malaria movement rates at high spatial and temporal resolutions. In most countries, censuses and household surveys are undertaken regularly, with the data being often made freely available at aggregated levels, meaning that the methods used here can be applied in other malarious regions. To expand the value of census and survey HPM data within regions such as East Africa, extracting and analyzing cross-border migration and travel-related data is important for a more comprehensive assessment of movement relevant for malaria control, elimination and drug resistance scenario planning [18], and future work will focus on this aspect. Developing sub-population surveys to obtain more detailed travel histories in high-risk migrant populations, such as durations of stay and access to healthcare within the 10–20 year old age group, would enable refined movement estimates within specific high-risk demographic groups to be made [19,30]. Specifically for migrant groups, respondent-driven sampling has been shown to be an effective technique for tracing frequency of travel to home locations [56].

## Conclusion

With funding expected to decline in the near future and the need for cost-effective intervention strategies [57], novel research methods that provide input to evidence-based decision-making are required. Here we have presented approaches that build on readily available datasets to quantify the variations in relative connectivity and mobility across countries and between demographic groupings. Through linkage with spatial malaria datasets, these outputs can be translated into quantitative estimates of malaria parasite movement routes, sources, sinks and rates, which are important for understanding transmission dynamics and designing effective and cost-efficient control strategies.

## Competing interests

The authors declare that they have no competing interests.

## Authors’ contributions

DKP did the literature search, identified datasets, carried out the analysis and wrote the first draft of the manuscript. AJG and ZH contributed to the analysis of the manuscript. DLS contributed to the analysis and review of the manuscript. VAA contributed to the data compilation. AMN and RWS contributed to the review of the manuscript. AJT contributed to the writing, analysis and review of the manuscript. All authors read and approved the final version of the manuscript.

## Supplementary Material

Additional file 1 East African datasets for malaria-relevant HPM for Kenya, Uganda and Tanzania. Click here for file

Additional file 2 Comparing connectivity similarities and differences between countries. Click here for file

Additional file 3 Short-term travel immigrant groups, stratified by rural/urban status and district of current residence in Kenya and regression models to statistically compare travel and bednet use between age groups, gender and migration status. Click here for file

Additional file 4 Kruskal-Wallis test results and boxplots to illustrate variations within age groups. Click here for file
