# Supplementary material for: The demographics of human and malaria movement and migration patterns in East Africa
Source: Malar J. 2013 Nov 5;12:397. doi: 10.1186/1475-2875-12-397 (PMC3829999; doi:10.1186/1475-2875-12-397)
Supplement: Additional file 1 — East African datasets for malaria-relevant HPM for Kenya, Uganda and Tanzania. [file 1475-2875-12-397-S1.pdf]

East African datasets for malaria-relevant HPM for Kenya, Uganda and Tanzania (most recent)

**Data type**	**Data set description**	**Malaria-relevant HPM data**
National Population and Housing Census	**Sample census datasets**<br>Kenya 2009^<br>Uganda 2002^<br>Tanzania 2002^	Selected variables for total census population: birth place, previous/current residence, demographic
	**Census microdata**<br>Kenya 1999*<br>Uganda 2002*<br>Tanzania 2002*	All census variables for random sample of census population: birth place, previous/current residence, age, gender, demographic
	**Bilateral migrant stock** for 226 census collecting countries*	Birth place, current residence
Household Surveys	**Household Budget Surveys**<br>Kenya Household Budget Survey 2004-05^	District of birth, village where respondent was raised, year of move, reason for move, cumulative time spent away in the last year, district of employment, demographic
	Uganda National Household Survey 2006*	HH type (IDP camps recorded), individual's residential status, duration of stay in HH in the last 12 months, reason for travel, resident elsewhere for > 6 months at a time in past year, time of most recent move, place of origin (name, urban status), reason for moving, other places of residence for >6 months, demographic
	Tanzania Household Budget Survey 2007*	Duration of residence in current location, demographic
	**Migration and Remittances Surveys** (African Migration Project)<br>Migration Household Survey in Kenya 2009*<br>Migration Household Survey in Uganda 2010*	Place of birth (rural/urban/other country), HH member living away, reason for living away, place of birth (rural/urban/other country) of the person living away, employment status, duration of stay in current location, previous place away for > 6 months (rural/urban/other country), time of move, time of return, reason for leaving, reason for destination choice, demographic
	**World Bank Living Standard Measurement Surveys (LSMS)**<br>Tanzania National Panel Survey 2008-09*	Cumulative time spent away in last 12 months, length of stay in current location, current/previous residence, reason for move, place of birth, demographic
	**Demographic Health Surveys (DHS)**<br>Kenya DHS 2008-2009*	Number of trips made in last 12 months, trips > 1 month in the last 12 months, demographic
	Uganda DHS 2006*<br>Tanzania DHS 2010*	Duration of stay in current location, previous residence (rural/urban), number of trips made in last 12 months, trips > 1 month in the last 12 months, demographic
	**Malaria Indicator Surveys (MIS)**<br>Kenya MIS 2010 (not yet available)<br>Tanzania MIS 2004 (2008, 2011)^	Number of trips away from home in the last 12 months, time spent away (>1 month) in the last 12 months, demographic, malaria-relevant factors

	**Labour Force Surveys**<br>Tanzania Integrated Labour Force Survey 2005/2006*<br><br>Tanzania Household Worker Survey 2004, 2005, 2006*	Duration in current residence, previous residence, reasons for move, children from HH living in another village/town (who they live with, what they do there, does respondent get in touch with HH, does respondent send money/goods), location of previous day's activity, demographic<br><br>Country of origin, city of birth, arrival date, home location (if different from current), region of residence at 6 years of age, location of current employment, employment-related travel, location whilst unemployed, demographic
Small-scale and sub-population surveys	Uganda Urban Inequities Survey 2006-07*<br><br>Northern Uganda Baseline Survey 2004*<br><br>Survey on Remittances to Uganda 2007*<br><br>Tanzania Core Welfare Indicator Survey 2003/2004*<br><br>Views of People Survey 2007*<br><br>MCC roads impact evaluation survey 2009*<br><br>A study of household travel in the Meru District of Kenya 1998*	Duration in current residence, reason for leaving previous house, place of birth, reason for 1st migration, year moved, demographic<br><br>HH type (IDP camps recorded), duration of stay in HH in the last 12 months, reason for travel, demographic<br><br>Duration of stay in HH in the last 12 months, years lived abroad, demographic<br><br>Duration away from HH in the last 12 months, demographic<br><br>Travel away from home, reason for travel, demographic<br><br>Previous visit, time of visit, time spent away in the last 12 months, expected returnees to HH, demographic<br><br>Main origins and destinations, reason for travel, mode of transport, demographic

*Data accessible free online (Table 1)

^Data available free upon request from Kenya National Bureau of Statisticshttp://www.knbs.or.ke/surveys.php; Uganda Bureau of Statistics www.ubos.org; National Bureau of Statistics Tanzania www.nbs.go.tz
