# Supplementary material for: The demographics of human and malaria movement and migration patterns in East Africa
Source: Malar J. 2013 Nov 5;12:397. doi: 10.1186/1475-2875-12-397 (PMC3829999; doi:10.1186/1475-2875-12-397)
Supplement: Additional file 2 — Comparing connectivity similarities and differences between countries. [file 1475-2875-12-397-S2.pdf]

Comparing connectivity similarities and differences between countries (un-weighted networks):

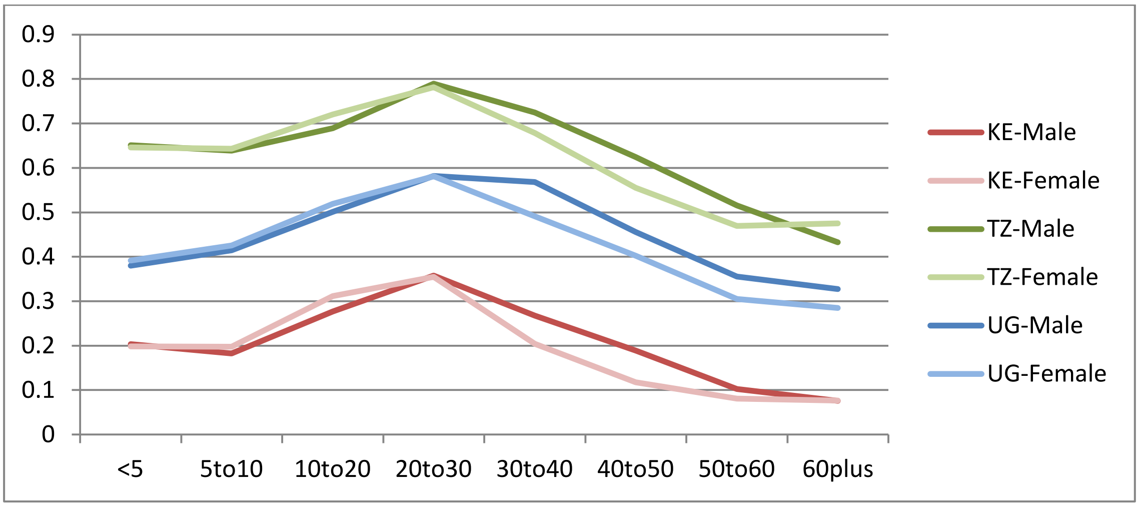

Density of HPM networks was different for each country because of the spatial-temporal definition of migration, based on the migration question asked. IPUMS migrant data were different based on time periods of migration and administrative boundary size between the datasets between countries. For Kenya, current residence was compared to previous residence one year ago, at a district level – second administrative boundary. For Uganda, current residence was compared to residence, not restricted to just one year, at a district level – second administrative boundary. For Tanzania, current residence was compared to residence 5 years ago, at a region level – first administrative boundary. Therefore direct comparisons between countries were not possible.

However, relative differences within each country could be made. For example, density of HPM networks for each country can be compared when stratified by age groups and gender (NB. density is a measure for un-weighted graphs so shows differences between connectivity in the 3 countries, between age groups and gender). Gender differences were seen in age-stratified HPM network densities for all three countries. Network densities were higher for older age groups in males compared to females. For younger age groups, differences between genders was smaller however female networks were more dense than male networks. Overall, male networks densities were higher than female network densities for all networks.
