# Supplementary material for: The demographics of human and malaria movement and migration patterns in East Africa
Source: Malar J. 2013 Nov 5;12:397. doi: 10.1186/1475-2875-12-397 (PMC3829999; doi:10.1186/1475-2875-12-397)
Supplement: Additional file 3 — Short-term travel immigrant groups, stratified by rural/urban status and district of current residence in Kenya and regression models to statistically compare travel and bednet use between age groups, gender and migration status. [file 1475-2875-12-397-S3.pdf]

Figure 1. Short term travel in migrant populations, stratified by rural urban status of current locations in Kenya (based on the Kenya Integrated Household Budget Survey - KIHBS)

Short term travel patterns were shown to vary between gender groups in rural and urban areas, with significant decreases in travel between >5 and 5-10 year old age groups for males from both rural and urban areas and females from rural areas. However, females from urban areas showed similar travel patterns throughout their lifespan, slightly increasing for older ages. Generally, in both rural and urban areas, male travel was higher in older age groups. Amongst children, highest travel was seen in youngest children, being likely to travel with their parents. 5-10 year children had lower rates of travel. However in older adolescent children in the 10-20 year age group, increases in travel amongst urban male migrants was seen. This may be a result of male migrant adolescents travelling back home to visit families during boarding school holidays.

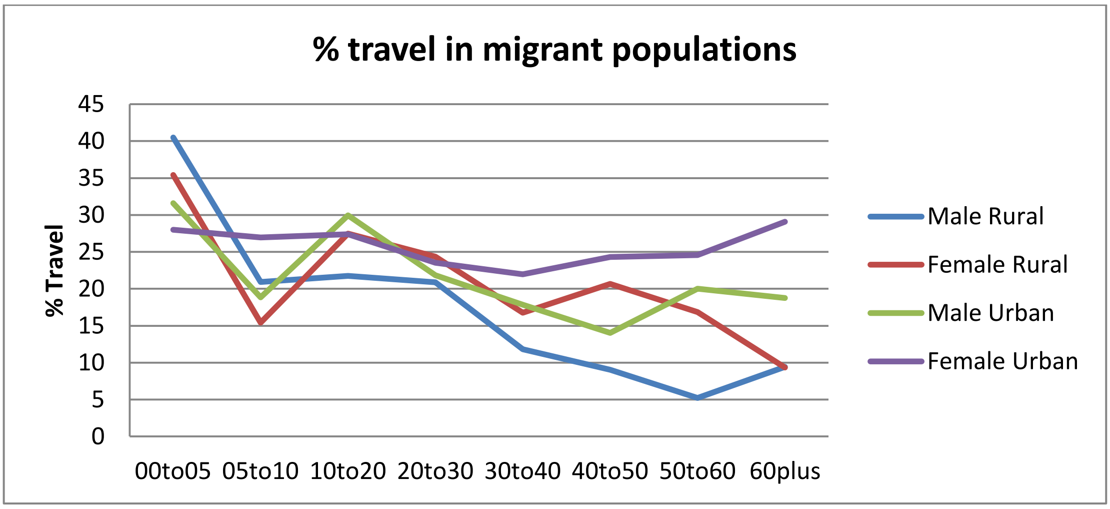

Figure 2. Short term travel in migrant groups, stratified by current district of residence in Kenya

Short term travel from low transmission to high transmission areas is a risk factor for malaria importation. This figure illustrates heterogeneity in short term travel patterns in migrants in different parts of Kenya.

(a) % migrants engaging in short term travel (b) Mean PfPR per district

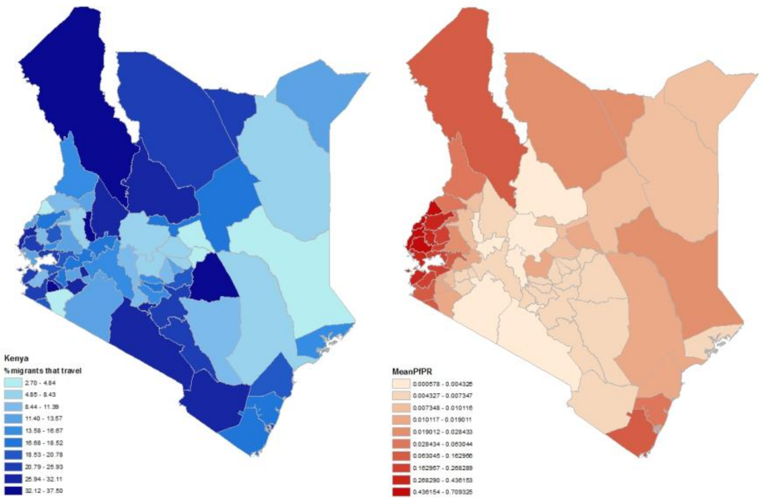

Regression Analysis results:

Dependent variables are travel and bed net use. Independent variables are age group, gender and migration status. Each independent variable is categorical.

Linear regression model 1:

Regress: Travel ~ AgeGroup + Gender + Migrant

Results:

Independent variable	Coefficient	Standard error	t	p-value
AgeGroup5-10	0.002318	0.004347	0.533	0.59
AgeGroup10-20	0.06046	0.003866	15.686	<0.05
AgeGroup20-30	0.121544	0.004226	28.763	<0.05
AgeGroup30-40	0.068702	0.004774	14.392	<0.05
AgeGroup40-50	0.062332	0.005310	11.739	<0.05
AgeGroup50-60	0.044329	0.006290	7.048	<0.05
AgeGroup60plus	0.025906	0.005991	4.324	<0.05
GenderFemale	-0.020596	0.002348	-8.773	<0.05
Migrants	0.310178	0.019912	15.578	<0.05

R-squared: 0.02165

Linear regression model 2:

Regress: Bednet ~ AgeGroup + Gender + Migrant

Results:

Independent variable	Coefficient	Standard error	t	p-value
AgeGroup5-10	-0.1027218	0.0066568	-15.431	<0.05
AgeGroup10-20	-0.1583671	0.0059205	-26.749	<0.05
AgeGroup20-30	-0.0426922	0.0064708	-6.598	<0.05
AgeGroup30-40	-0.0004049	0.0073097	-0.055	0.96
AgeGroup40-50	-0.0574785	0.0081311	-7.069	<0.05
AgeGroup50-60	-0.1139525	0.0096313	-11.831	<0.05
AgeGroup60plus	-0.174645	0.0091736	-19.038	<0.05
GenderFemale	0.0259087	0.0035949	7.207	<0.05
Migrants	0.1020865	0.0304905	3.348	<0.05

Reference groups = AgeGroup<5, GenderMale and Non-migrants

R-squared: 0.02017
