# Supplementary material for: The demographics of human and malaria movement and migration patterns in East Africa
Source: Malar J. 2013 Nov 5;12:397. doi: 10.1186/1475-2875-12-397 (PMC3829999; doi:10.1186/1475-2875-12-397)

A. Kruskal-Wallis test results

1. Migration network connectivity (comparing mean network degree for each age, gender, rural-urban stratified network):

Comparing mean degree between the networks using the Kruskal-Wallis test showed that Ugandan rural and urban females were significantly different and Ugandan urban females and rural males were significantly different. All other gender-rural-urban stratified networks were not statistically different from each other. Statistical comparisons were also made for age groups. In Kenya, the 10-20 year age group was statistically different from the 50-60 and 60 plus age groups, and the 20-30 year age group was statistically different from the 50-60 and 60 plus age groups. In Tanzania, statistically significant differences were seen between the 20-30 year age group and the 50-60 and 60 plus age groups. In Uganda, no statistically significant differences between age groups were seen for connectivity.

2. Migration magnitude (comparing mean network strength for each age, gender, rural-urban stratified network):

HPM flow networks showed no statistical differences between gender-rural-urban groups for any country. However, differences in magnitudes of flows were seen between age groups in Kenya and Tanzania, between 10-20 and 50-60 year age groups, 10-20 and 60 plus age groups, 20-30 and 50-60 year age groups and 20-30 and 60 plus age groups.

3. Relative malaria movement magnitude (comparing mean network strength for each age, gender, rural-urban stratified malaria-weighted network):

Comparing HPM flow networks showed no statistically significant differences between gender-rural-urban groups for any country. However, differences in magnitudes of flows were seen between age groups. In Uganda, statistically significant differences in malaria movement were seen between the 10-20 and 50-60 year age groups, 10-20 and 60 plus age groups, 20-30 and 50-60 year age groups and 20-30 and 60 plus age groups. In Kenya, statistically significant differences in malaria movement were seen between the 10-20 and 50-60 year age groups, 10-20 and 60 plus age groups, and 20-30 and 50-60 year age groups. In Tanzania, statistically significant differences in malaria movement were seen 5-10 and 60 plus age groups, 10-20 and 60 plus age groups, 20-30 and 50-60 year age groups and 20-30 and 60 plus age groups.

B. Boxplots

1. **Within age group variation in connectivity, HPM flow magnitudes and malaria movement in Kenya, Tanzania and Uganda**

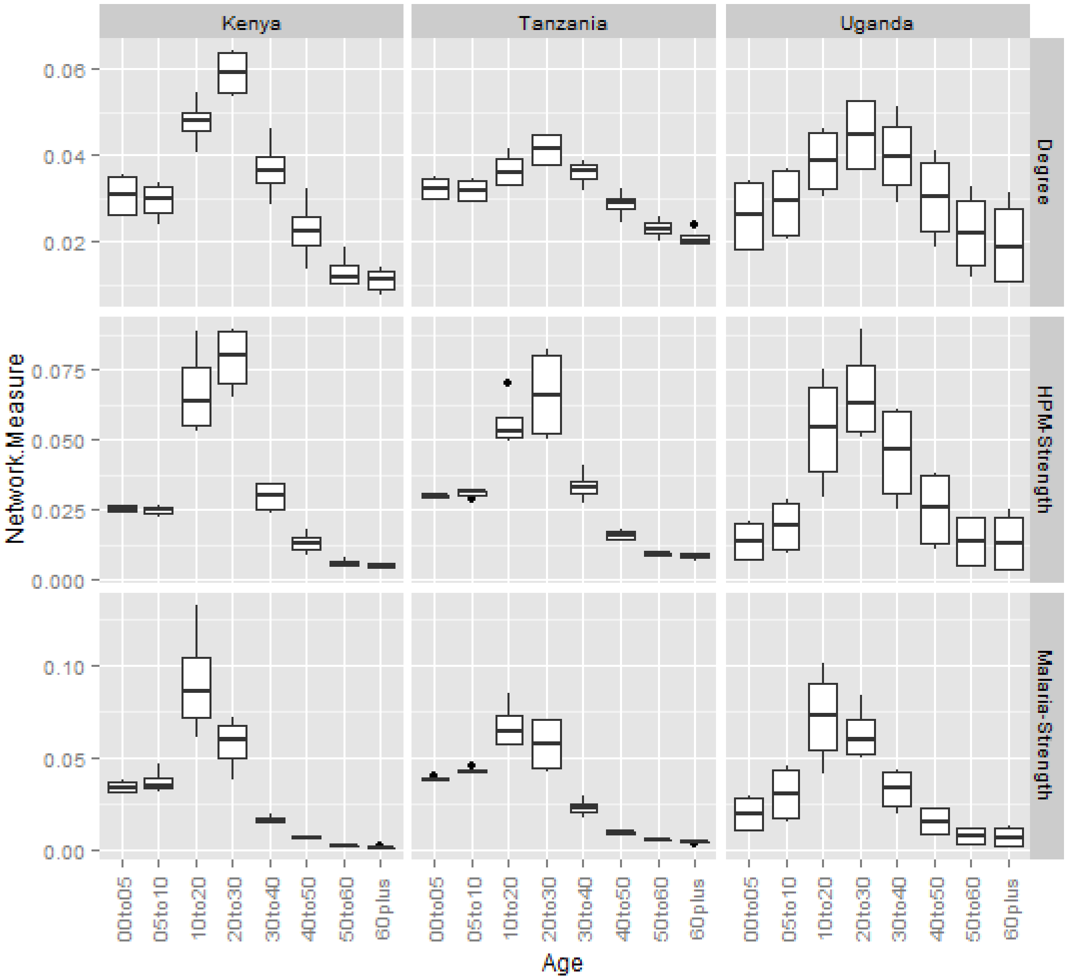

2. **Within age group variation in malaria movement, stratified by gender, for Kenya, Tanzania and Uganda**

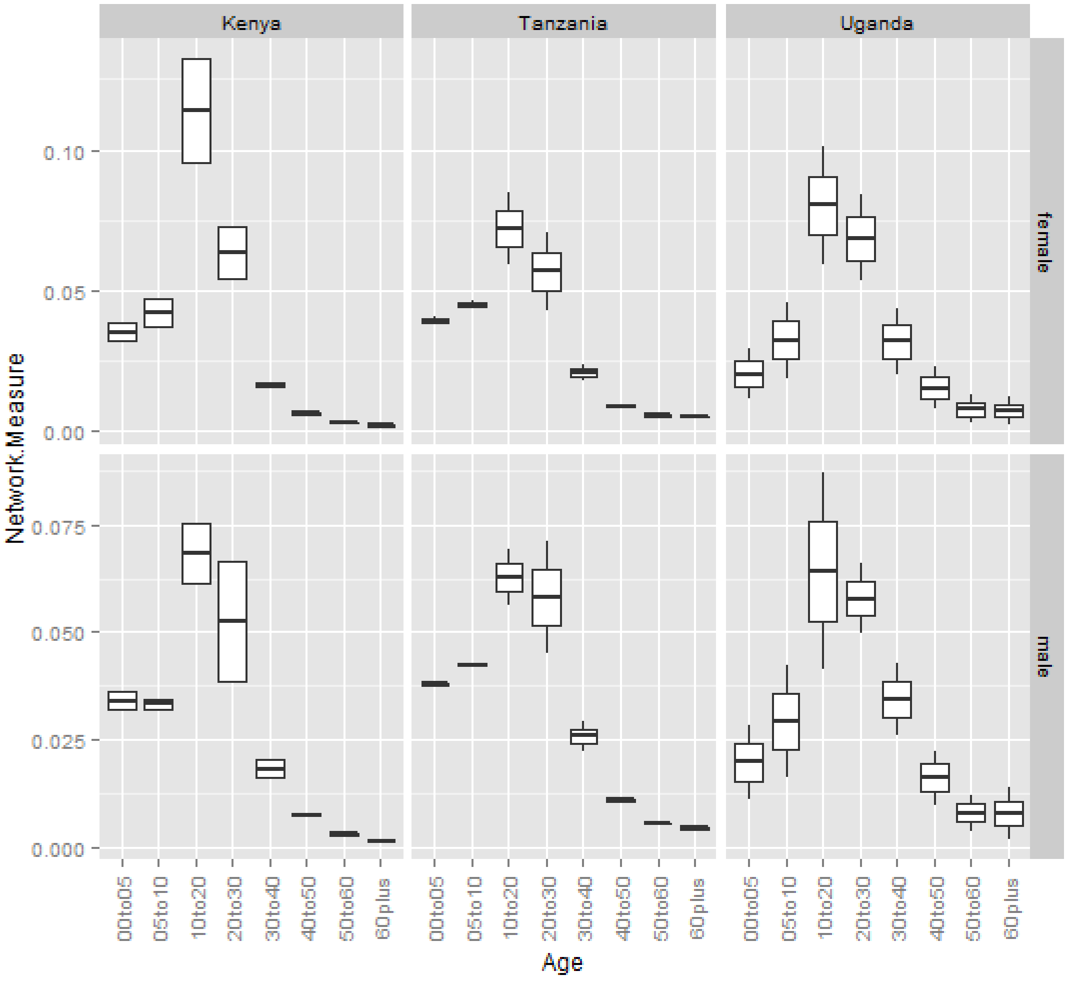

3. **Within age group variation in malaria movement, stratified by rural-urban status, for Kenya, Tanzania and Uganda**

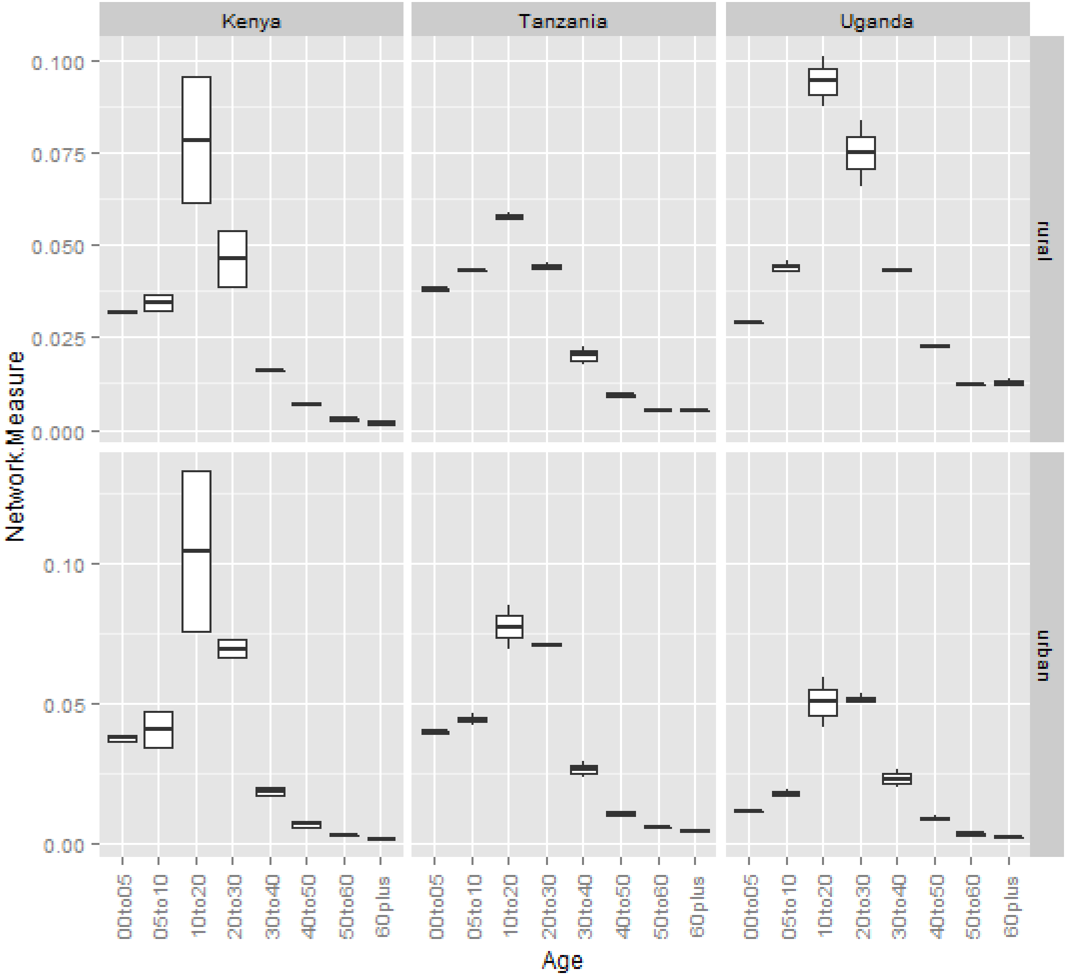

Supplement: Additional file 4 — Kruskal-Wallis test results and boxplots to illustrate variations within age groups. [file 1475-2875-12-397-S4.pdf]
